# Supplementary material for: High-resolution spectroscopy of buffer-gas-cooled phthalocyanine
Source: Commun Chem. 2022 Nov 29;5:161. doi: 10.1038/s42004-022-00783-4 (PMC9814875; doi:10.1038/s42004-022-00783-4)
Supplement: Supplementary file 1 — Supplementary Information [file 42004_2022_783_MOESM1_ESM.pdf]

Supplementary Information on “High-resolution spectroscopy of buffer-gas-cooled phthalocyanine”

Yuki Miyamoto, Reo Tobaru, Yuiki Takahashi, Ayami Hiramoto, Kana Iwakuni, Susumu Kuma, Katsunari Enomoto, and Masaaki Baba

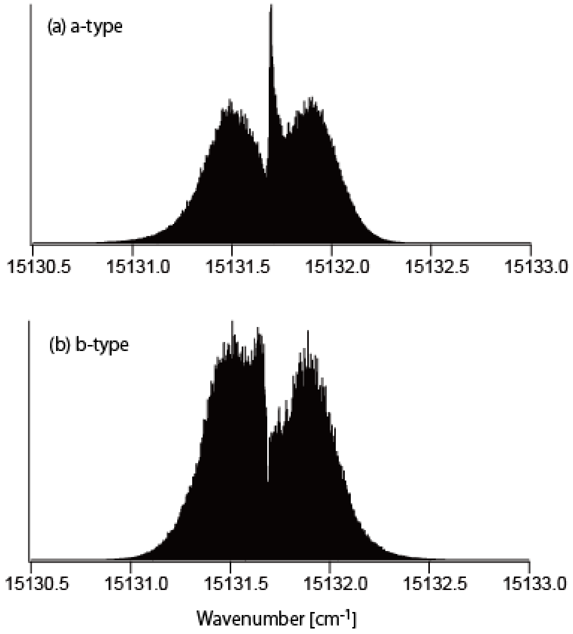

Supplementary Fig. 1 Simulated spectra for a- and b-type $S_1 \leftarrow S_0$ transition of free base phthalocyanine. Simulations were performed by PGOPHER [1] assuming (a) a-type transition and (b) b-type transition. The simulation was performed with D_{2h} symmetry. The irreducible representation of the ground state is A_g while those of the excited state are B_{3u} (a-type) or B_{2u} (b-type). The rotational and translational temperatures are 10 K. Rotational constants are set as follows: $A_{ground} = 0.002991$, $B_{ground} = 0.002986$, $C_{ground} = 0.001494$, $A_{excited} = 0.002994$, $B_{excited} = 0.002973$, and $C_{excited} = 0.001497$ (cm^{-1}). It should be noted that these values are inconclusive. Although a more detailed discussion of the simulation and rotational constants will be reported in a separate paper, the results suggest that the observed transition is a b-type transition.

Supplementary References

[1] Western, C. M., PGOPHER: A program for simulating rotational, vibrational and electronic spectra, *J. Quant. Spectrosc. Rad. Trans.* **186**, 221 (2017).
